# Real-time AIoT platform for monitoring and prediction of air quality in Southwestern Morocco

**DOI:** 10.1371/journal.pone.0307214

**Published:** 2024-08-22

**Authors:** Abdellatif Bekkar, Badr Hssina, Najib ABEKIRI, Samira Douzi, Khadija Douzi

**Affiliations:** 1 LIM Laboratory, Faculty of Sciences and Technics Hassan II University of Casablanca, Mohammedia, Morocco; 2 Faculty of Medicine and Pharmacy, Mohammed V University in Rabat, Rabat, Morocco; 3 Higher School of Technology, University Ibn Zohr, Agadir, Morocco; Chiang Mai University, THAILAND

## Abstract

Urbanization and industrialization have led to a significant increase in air pollution, posing a severe environmental and public health threat. Accurate forecasting of air quality is crucial for policymakers to implement effective interventions. This study presents a novel AIoT platform specifically designed for PM_2.5_ monitoring in Southwestern Morocco. The platform utilizes low-cost sensors to collect air quality data, transmitted via WiFi/3G for analysis and prediction on a central server. We focused on identifying optimal features for PM_2.5_ prediction using Minimum Redundancy Maximum Relevance (mRMR) and LightGBM Recursive Feature Elimination (LightGBM-RFE) techniques. Furthermore, Bayesian optimization was employed to fine-tune hyperparameters of popular machine learning models for the most accurate PM_2.5_ concentration forecasts. Model performance was evaluated using Root Mean Square Error (RMSE), Mean Absolute Error (MAE), and the coefficient of determination (R^2^). Our results demonstrate that the LightGBM model achieved superior performance in PM_2.5_ prediction, with a significant reduction in RMSE compared to other evaluated models. This study highlights the potential of AIoT platforms coupled with advanced feature selection and hyperparameter optimization for effective air quality monitoring and forecasting.

## 1 Introduction

The rapid pace of industrialization and urbanization has significantly reshaped the global landscape. According to the United Nations, over 50% of the world’s population now resides in urban areas, a figure expected to rise in the coming years [1]. While urbanization has driven economic growth and improved living standards, it has also introduced significant challenges, notably in transportation, healthcare, and air pollution [2]. To address these issues, the concept of smart cities has emerged, leveraging advanced information and communication technologies to promote sustainability and enhance quality of life.

One critical challenge in urban areas is air pollution, driven by the extensive use of fossil fuel-based vehicles and industrial equipment. Harmful gases and particulate matter, including carbon oxides (COx—CO and CO_2_), nitrogen oxides (NOx—NO and NO_2_), sulfur oxides (SOx—SO_2_, SO_3_, and SO_4_), and particulate matter (PM_10_ and PM_2.5_), pose serious health risks due to their small size and ability to penetrate respiratory systems. The World Health Organization (WHO) attributes approximately seven million deaths annually to air pollution, with about 90% of the global population exposed to polluted air [3]. Health impacts include respiratory issues, premature mortality, and increased hospital admissions for cardiovascular and pulmonary diseases [4, 5]. Additionally, prolonged exposure to air pollutants can damage vegetation, affecting agricultural productivity and natural ecosystems [6].

Particulate matter (PM), particularly particles smaller than 2.5 microns (PM_2.5_), has been the focus of recent studies due to its severe health implications. These fine particles can penetrate deep into lung tissue, causing respiratory diseases, asthma, cardiovascular problems, and even mortality [7–9]. Moreover, evidence suggests that PM pollution may facilitate the spread of viruses like SARS-CoV-2 [10]. Accurate assessment and prediction of PM_2.5_ levels are therefore crucial for effective air pollution management.

Traditional urban air quality monitoring relies on fixed monitoring stations, which are expensive to install and maintain, with each station costing at least 10,000 USD excluding installation and maintenance expenses [11]. Despite their high cost, the distribution of these stations is often inadequate, even in developed countries [12]. For instance, Morocco, with an area of 710,850 km^2^ and a population of over 38 million, has only 29 regulatory air quality monitoring stations, indicating insufficient coverage [13]. In metropolitan areas, atmospheric pollutant dispersion can vary significantly over short distances (< 1 km) [12]. This variability, driven by unevenly distributed emission sources and complex urban dispersion processes, makes conventional stationary monitoring stations site-specific and often insufficient for capturing real-time air quality variations.

To address these challenges, there has been a shift towards using small, affordable sensing devices, or IoT units. Deploying numerous low-cost air sensors that provide frequent data updates is now feasible [14]. These sensors are significantly cheaper than traditional fixed monitoring stations, with costs ranging from 100 to 2500 USD [15]. The integration of IoT technology with machine learning algorithms presents a promising approach to enhance air quality management. Machine learning techniques, such as Support Vector Regression (SVR), Gradient Boosting, and LightGBM, have demonstrated effectiveness in forecasting PM_2.5_ concentrations using various environmental and meteorological data [16]. Mampitiya et al. showcased the high effectiveness of LightGBM in forecasting PM_10_ levels in urban areas of Sri Lanka, achieving near-perfect accuracy metrics [17]. Additionally, integrating machine learning with remote sensing data, as explored by Rostami et al., enhances the precision of environmental assessments [18]. Bekkar et al. highlighted the effectiveness of deep learning approaches in predicting air pollution in smart cities [19]. The synergy between advanced algorithms and IoT data collection systems offers a powerful tool for real-time monitoring and prediction, enabling proactive measures to mitigate pollution impacts.

Despite significant advances in air quality monitoring and prediction, substantial gaps remain. Most studies focus on highly industrialized regions, neglecting urban areas in developing countries like Morocco. Existing models often fail to integrate low-cost IoT sensors for real-time data collection and prediction, limiting their applicability in resource-constrained settings.

This study aims to develop an efficient, AI-integrated air pollution monitoring system tailored for smart urban environments, capable of providing timely alerts and accurate predictions of pollution levels to mitigate health impacts. The novelty of this work lies in integrating low-cost IoT sensors with advanced machine learning techniques, enabling real-time air quality monitoring and prediction in resource-limited settings. The use of feature selection methods like mRMR and LightGBM-RFE, combined with Bayesian optimization for hyperparameter tuning, ensures high accuracy in PM_2.5_ predictions. This approach provides a scalable and cost-effective solution for air quality monitoring in developing countries.

The remainder of this paper is structured as follows: Section 2 reviews the related work on air quality monitoring and prediction. Section 3 expands on the IoT platform devised for data collection. Section 4 describes our approach to sensor implementation. Section 5 presents preliminary results and corresponding analysis. Section 6 discusses the utilization of machine learning algorithms for air quality forecasting. Finally, Section 7 offers conclusions and potential avenues for future advancements.

## 2 Related work

The increasing urgency for accurate and comprehensive air quality monitoring has intensified due to rapid industrialization and urbanization. Traditional air quality monitoring methods rely on fixed monitoring stations equipped with high-precision instruments such as gas analyzers and particulate matter sensors. These stations, often managed by government agencies, provide reliable measurements of pollutants like NO_2_, SO_2_, CO, O_3_, and PM_2.5_ [20]. However, the high costs associated with their setup, calibration, and maintenance restrict their deployment, resulting in limited spatial coverage and insufficient data to capture localized pollution events or micro-scale air quality variations [21]. To address these limitations, researchers have explored alternative methods that offer greater flexibility and cost-efficiency.

The advent of the Internet of Things (IoT) has introduced a paradigm shift in air quality monitoring by enabling the deployment of low-cost sensors that can be distributed extensively. IoT-based systems utilize sensors to measure various pollutants and transmit data in real-time to centralized databases for analysis. These systems offer several advantages, including reduced costs, enhanced spatial coverage, and the ability to provide high-resolution temporal data [22]. IoT sensors can be integrated into stationary nodes, mobile units, and wearable devices, allowing for flexible and comprehensive monitoring solutions. For example, Kumar et al. demonstrated the effectiveness of deploying a dense network of low-cost IoT sensors across urban areas, providing detailed spatial and temporal pollution data [23]. The deployment of IoT sensors in smart cities has shown promising results in improving air quality monitoring and enabling proactive pollution mitigation measures [24, 25]. Additionally, IoT-based systems facilitate the development of advanced data analytics and machine learning models to predict pollution levels, identify pollution sources, and inform policy-making, while also raising public awareness by providing real-time air quality information through mobile apps and web platforms [26].

Machine learning techniques have been increasingly applied to predict air quality, leveraging historical data to forecast future pollution levels. Supervised learning models such as Support Vector Regression (SVR), Random Forests (RF), and Gradient Boosting Machines (GBM) have shown considerable promise. SVR has been widely used for its robustness in handling non-linear relationships between input variables and air quality indices. For instance, Chen et al. demonstrated the effectiveness of SVR in predicting PM_2.5_ concentrations with high accuracy, outperforming traditional statistical methods [27]. Random Forests, known for their ability to handle large datasets and complex interactions, have also been employed successfully. Jiang et al. utilized RF to predict air quality levels and identified significant predictors among meteorological variables and pollutant concentrations [28]. Gradient Boosting, another powerful ensemble technique, combines multiple weak predictive models to form a strong predictor. Studies like those by Li et al. have shown that GBM can effectively model the temporal and spatial variations in air pollution, achieving superior performance compared to individual models [29].

Ensemble methods, which integrate multiple learning algorithms to improve predictive performance, have gained traction in air quality prediction. Techniques such as Light Gradient Boosting Machine (LightGBM) are particularly noted for their efficiency and accuracy. LightGBM, a variant of GBM, optimizes the training process by focusing on gradient-based one-side sampling and exclusive feature bundling, making it suitable for large-scale data [30]. Comparative studies have highlighted the advantages of ensemble methods over single models. For example, Zhang et al. found that LightGBM consistently outperformed other methods in terms of prediction accuracy and computational efficiency [31]. These findings underscore the potential of ensemble approaches in enhancing the reliability and precision of air quality forecasts.

In Morocco, several studies have focused on understanding the seasonal variations and meteorological influences on air quality. Bounakhla et al. provided an overview of PM10, PM2.5, and black carbon (BC) and their relationships with meteorological variables in Kenitra, Morocco. Their research highlighted significant seasonal variations in pollutant concentrations and the influence of temperature, humidity, and wind speed on these pollutants [32]. This aligns with the findings of Sbai et al., who investigated the response of atmospheric pollutants to emission reduction and meteorological factors during the COVID-19 lockdown in northern Morocco. Their study emphasized the impact of meteorological conditions on secondary air pollutants like PM2.5 [33].

The integration of low-cost sensors and IoT technologies has been explored by Fahim et al. in developing a smart weather monitoring station for air quality assessment. Their system uses a fuzzy inference model and MQTT protocol to provide accurate and real-time air quality data, crucial for effective environmental management [34]. Additionally, a systematic review by Bouchriti et al. on the health impacts of outdoor air pollution in Morocco highlights the significant health issues caused by pollutants like PM10 and PM2.5, underscoring the need for continuous monitoring and predictive modeling to mitigate these effects [35].

Local implementations of air quality monitoring systems in Moroccan cities have demonstrated the potential for low-cost, high-efficiency solutions. For example, deploying an IoT-based air quality monitoring system in an urban area of Morocco showcased the effectiveness of real-time data collection and analysis in identifying pollution hotspots [36]. Similarly, using a geographic information system (GIS) to provide real-time air quality information to citizens has proven effective in raising awareness and promoting environmental health [37].

Despite significant advancements, gaps remain in the research on air quality monitoring and prediction in Morocco. There is a need for extensive research on the health and economic impacts of air pollution, improved air quality modeling, and a broader pollutant focus beyond just regulated ones. Developing comprehensive datasets, such as the MOREAIR dataset, which includes temporal, geographical, and air-quality measurements, can provide a richer context for understanding air pollution’s impact [38].

Our study builds upon this body of work by integrating low-cost IoT sensors, machine learning models, and real-time data analytics specifically tailored for air quality monitoring in Morocco. By leveraging these technologies, our research aims to achieve accurate air quality predictions and provide valuable insights into the health impacts of air pollution. The novel integration of AIoT platforms with advanced feature selection and hyperparameter optimization techniques, such as mRMR and LightGBM-RFE, ensures high accuracy in PM_2.5_ predictions. This approach addresses the challenges identified in previous studies and offers a scalable and cost-effective solution for air quality monitoring in developing countries.

## 3 IoT platform / IoT monitoring system

**Fig 1:**
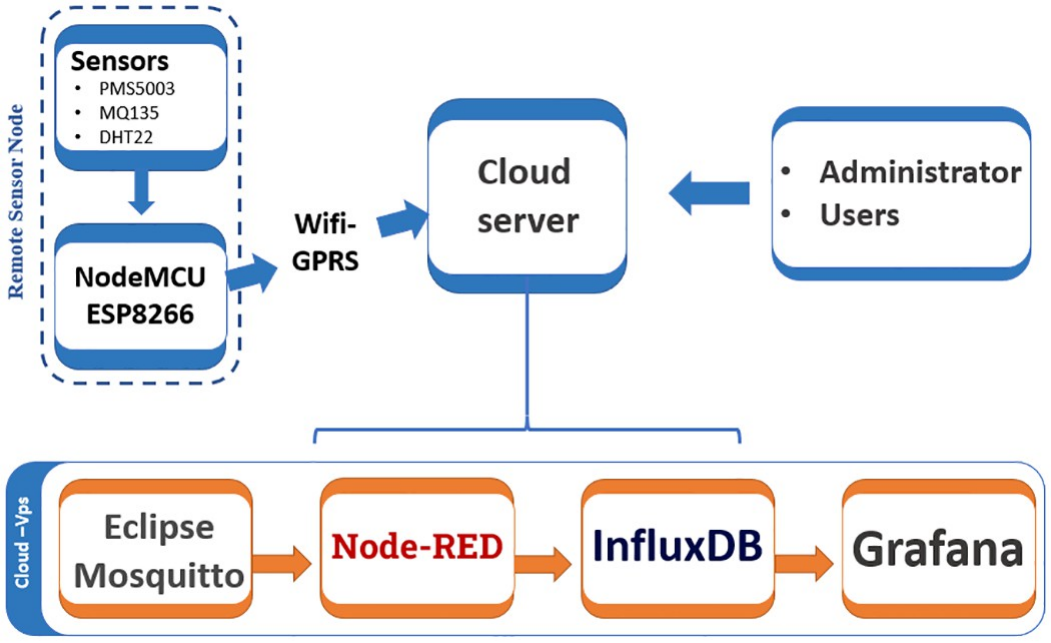
Workflow diagram of the IoT monitoring system.

This study presents an innovative and cost-effective IoT-based monitoring system designed to accurately measure fine particulate matter, specifically PM_2.5_ and PM_10_, in micrograms per cubic meter (*μg*/*m*^3^). This system enables wireless data transfer from multiple geographic locations to a centralized cloud server, facilitating comprehensive storage, analysis, and real-time visualization. The IoT monitoring system consists of two core components: Remote Sensor Nodes (RSN) and a Cloud Server (CS), as depicted in Fig 1. The RSNs collect environmental data, which is then transmitted to the CS for aggregation, processing, and visualization. This architecture supports efficient, scalable monitoring and provides valuable insights into environmental conditions, aiding data-driven decision-making for urban planning and public health management.

### 3.1 Remote Sensor Nodes

**Fig 2:**
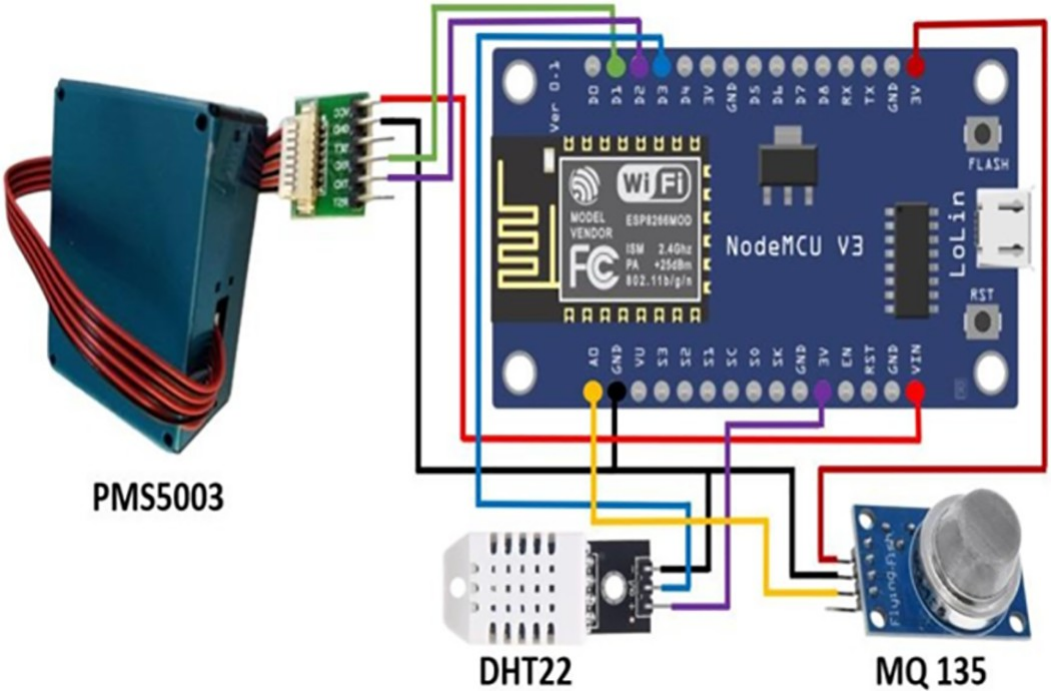
Schematic diagram of the wireless environmental monitoring device integrating PMS5003, DHT22, and MQ135 sensors with NodeMCU V3 (ESP8266).

The Remote Sensor Nodes (RSN) are crucial for the IoT monitoring system, enabling wireless environmental data collection and transmission. Each RSN integrates various sensors with an ESP8266 microcontroller, known for its cost-effective and reliable wireless communication capabilities [39] (see Fig 2).

#### 3.1.1 DHT22 sensor

The DHT22 measures temperature and humidity with high accuracy (±0.5°*C* for temperature and ±2% rH for humidity) and provides a digital output [40].

#### 3.1.2 PMS5003 sensor

The PMS5003 detects PM_2.5_ and PM_10_ using a fan mechanism, offering near-accurate results and affordability (around 15 USD) [41].

#### 3.1.3 MQ135 gas sensor

The MQ135 detects harmful gases like ammonia, sulfur dioxide, and benzene. Proper calibration ensures accurate measurements despite resistance variability [42].

#### 3.1.4 Data transmission

Data from the sensors is digitized and transmitted wirelessly to the Cloud Server via Wi-Fi or GPRS, using the lightweight and reliable MQTT protocol.

The integration of these components within the RSN provides a robust, scalable solution for real-time air quality monitoring, enhancing urban planning and public health management.

### 3.2 Server coordinator

This section outlines the robust communication framework designed to collect and present real-time environmental data from various Remote Sensor Nodes (RSNs) to a centralized Cloud Server (CS). The framework employs Mosquitto MQTT [43], Node-RED, InfluxDB, and Grafana, ensuring seamless data transmission, processing, and visualization.

#### 3.2.1 MQTT protocol

The Message Queuing Telemetry Transport (MQTT) protocol [44] is ideal for lightweight messaging in IoT applications due to its simplicity, efficiency, and reliability. Client devices (RSNs) connect to a centralized broker on a cloud server, publishing or subscribing to messages using designated topics.

#### 3.2.2 Node-RED

Node-RED [45], an open-source platform based on Node.js, connects devices, APIs, and services. It processes MQTT-transmitted sensor data, extracting and reassembling measurements for storage or transmission to InfluxDB.

#### 3.2.3 InfluxDB

InfluxDB [46], an open-source time-series database optimized for IoT applications, handles large volumes of time-based data efficiently. It integrates seamlessly with Python for robust data retrieval and storage.

#### 3.2.4 Grafana

Grafana [47] is an open-source application for data analysis and visualization, supporting various data sources including InfluxDB. It offers extensive graphical representations and can send notifications based on predefined criteria for real-time monitoring.

This integrated framework ensures reliable and efficient data management, supporting the IoT monitoring system’s goal of providing accurate, real-time insights into air quality and environmental conditions, thereby facilitating informed urban planning and public health management.

## 4 Experiment

### 4.1 Deployment strategy

Ait Melloul, a municipality in southwestern Morocco, spans 40 km^2^ and has a population of over 171,847. Located about 15 km from Agadir, it experiences a semi-arid to arid climate influenced by the Atlas Mountains, the Atlantic coast, and the desert. According to [48], aridity increases from west to east. The average annual precipitation is around 260 mm, with temperatures ranging from a high of 27°*C* in August to a low of 11°*C* in January. Humidity levels range between 32% and 85%, and prevailing winds from the west-northwest blow at speeds of 0.1 to 3.3 m/s. These conditions can cause temperature inversions, trapping pollutants in the lower atmosphere, making the region vulnerable to climate change impacts.

**Fig 3:**
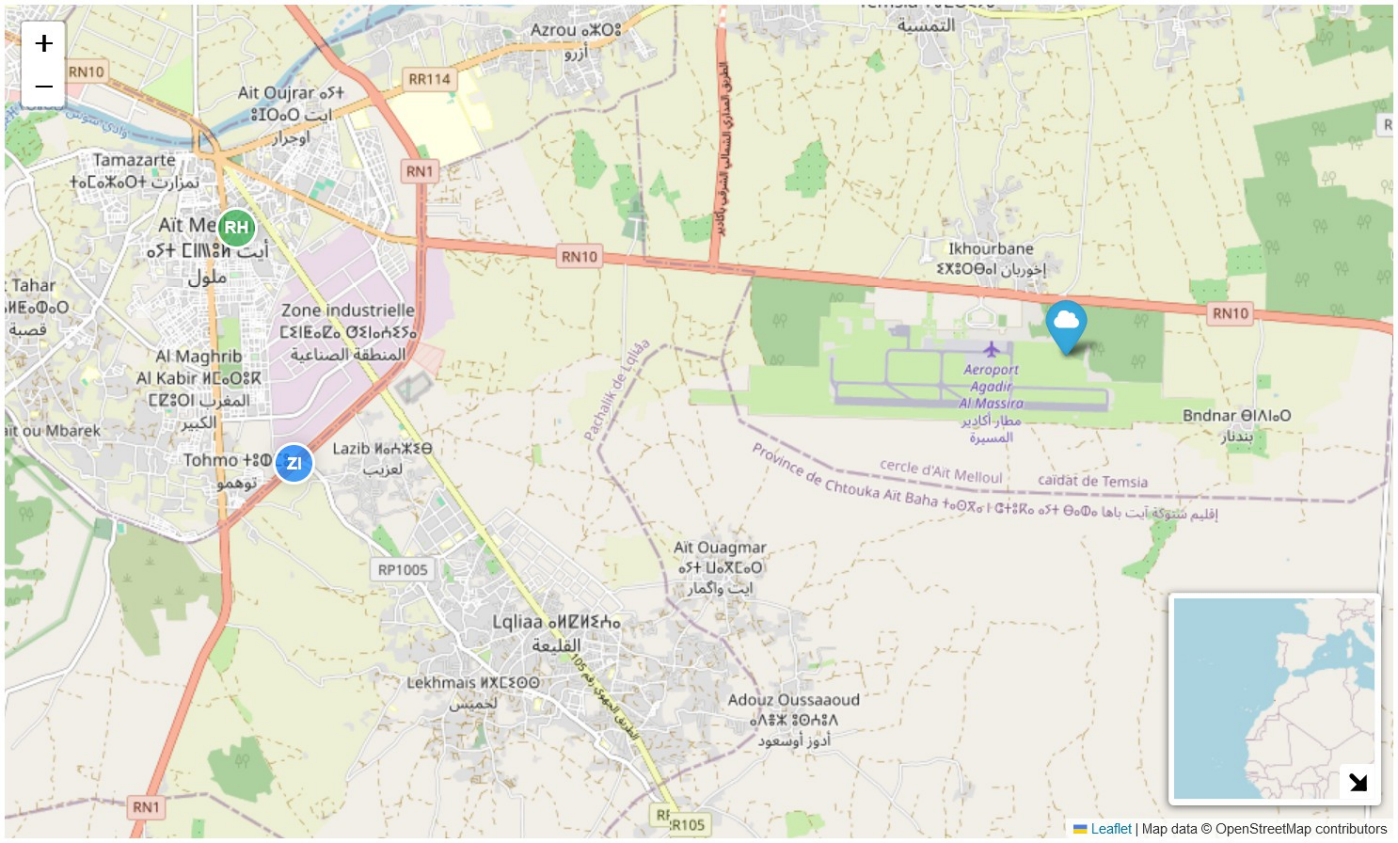
Location of the experimental facility in Ait Melloul. Map data (C) OpenStreetMap contributors, under the Open Database License (ODbL). This map was created using the folium library in Python.

Two locations within Ait Melloul were selected for their proximity to vehicular traffic and industrial facilities, as shown in Fig 3. The first site, S1, is in the Industrial Zone, near the intersection of RN1 and the expressway to Agadir Al Massira International Airport. This area experiences heavy vehicular traffic, including large trucks. The second site, S2, is situated between two heavily trafficked lanes of RN1.

No specific permits were required for this study. Air quality data was collected using a low-cost, non-invasive monitoring system. Local authorities confirmed that no specific permits were necessary for this type of data collection and its use in research.

### 4.2 Exploratory data analysis

Data collection occurred from October 2022 to February 2023, resulting in 3504 hourly data points, providing high-resolution temporal data. Weather data were obtained via the Visual Crossing Weather API [49], with the GMAD weather station located at coordinates (30.33, -9.4). This station was chosen due to its proximity within a 10 km radius of the pollutant monitoring station. The data were received in CSV format, comprising 5 pollutant variables and 13 meteorological variables.

The dataset collected by our platform is crucial as it is specifically gathered from Southwestern Morocco, ensuring relevance to local air pollution challenges. It includes high-resolution, real-time measurements taken hourly, capturing temporal variations in pollutant levels. Our AIoT platform is tailored to collect comprehensive environmental data, demonstrating the feasibility and effectiveness of low-cost IoT sensors in resource-limited settings. This innovative integration of sensors and machine learning offers a practical solution for real-time air quality monitoring and prediction, providing a valuable baseline for future research and public health benefits.

The statistical characteristics of the data collected from the monitoring points in Ait Melloul are presented in Table 1. The analysis of PM_2.5_ can be conducted in temporal and spatial dimensions, where prior concentrations of PM_2.5_ may impact subsequent measurements. The PM_2.5_ concentration is influenced by various factors, including the interaction between pollutants and meteorological variables, such as wind speed and direction.

**Table 1:** Basic statistics of pollutants and meteorological data of monitoring points in industrial zone.

Parameter	Symbol	Unit	Count	Min	Max	Mean	Std
Particulate Matter 2.5	PM2.5	*μg*/*m*^3^	3251	0	133	12.53	11.78
Particulate Matter 10	PM10	*μg*/*m*^3^	3251	0	326	16.12	16.08
Carbon Monoxide	CO	ppm	3252	0.28	160	7.72	13.74
Carbon Dioxide	CO2	ppm	3252	0.16	1021	23.64	66.98
Relative Humidity	RH	%	3252	16.5	99.9	65.63	20.46
Relative Temperature	RT	%	3252	7.78	41	20.92	5.76
Temperature	temp	°*C*	3504	4.10	39	18.37	6.20
Feels Like	feelslike	°*C*	3504	1.80	36.3	18.10	6.10
Dew Point	dew	°*C*	3504	-12.6	21	7.82	6.12
Humidity	humidity	%	3504	3.92	100	58.38	27.48
Precipitation	precip	mm	3504	0	19	0.03	0.48
Wind Gust	windgust	kph	3504	1.10	76	21.12	12.72
Wind Speed	windspeed	kph	3504	0	55.9	13.24	7.93
Wind Direction	winddir	degrees	3504	0	360	142.77	93.70
Sea Level Pressure	pressure	mb	3504	1006	1029.3	1018.59	4.37
Cloud Cover	cloudcover	%	3504	0	100	32.22	36.45
Visibility	visibility	km	3504	0	24.1	9.20	1.50

This table presents the basic statistical characteristics of pollutants and meteorological data collected at monitoring points in the Industrial Zone of Ait Melloul.

Through an examination of the interplay among PM_2.5_, atmospheric pollutants, and meteorological conditions, a greater understanding of the diverse influences on PM_2.5_ can be attained. The PM_2.5_ concentration at target stations can be affected by changes in wind direction and airflow from surrounding stations when viewed through a spatial lens. Therefore, it is imperative to consider the spatial latitude correlation when forecasting, mitigating, and managing PM_2.5_ pollution.

**Fig 4:**
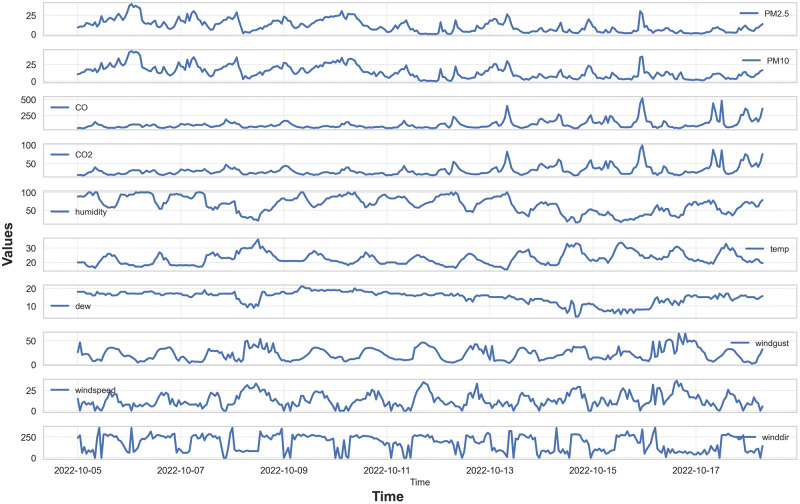
Temporal fluctuations of pollution and meteorological parameters. The data were observed at station 1 during the initial days of October.

Fig 4 illustrates the temporal fluctuations of pollution and meteorological parameters recorded at station 1 during the initial days of October. The variation patterns of PM_10_ and PM_2.5_ concentrations are closely aligned, suggesting a significant influence of PM_10_ on PM_2.5_ levels. Similar trends are observed in the fluctuations of carbon monoxide (CO) and carbon dioxide (CO_2_).

Notably, local maxima in humidity, dew point, wind speed, and wind direction coincide with a gradual decrease in PM_2.5_ concentrations. Elevated relative humidity enhances the adsorption capacity of PM_2.5_ particles, leading to the condensation of moisture-rich fine particles and the formation of larger particles that settle out of the air, thus reducing PM_2.5_ concentrations. Additionally, increased wind speeds aid in the dispersion of particles, potentially contributing to lower PM_2.5_ levels. Initial analysis indicates a negative correlation between PM_2.5_ concentrations and factors such as humidity, dew point, wind speed, and wind direction [50, 51].

These findings underscore the complex interactions between pollutant levels and meteorological conditions, highlighting the critical role of these factors in air quality management in industrial zones.

**Fig 5:**
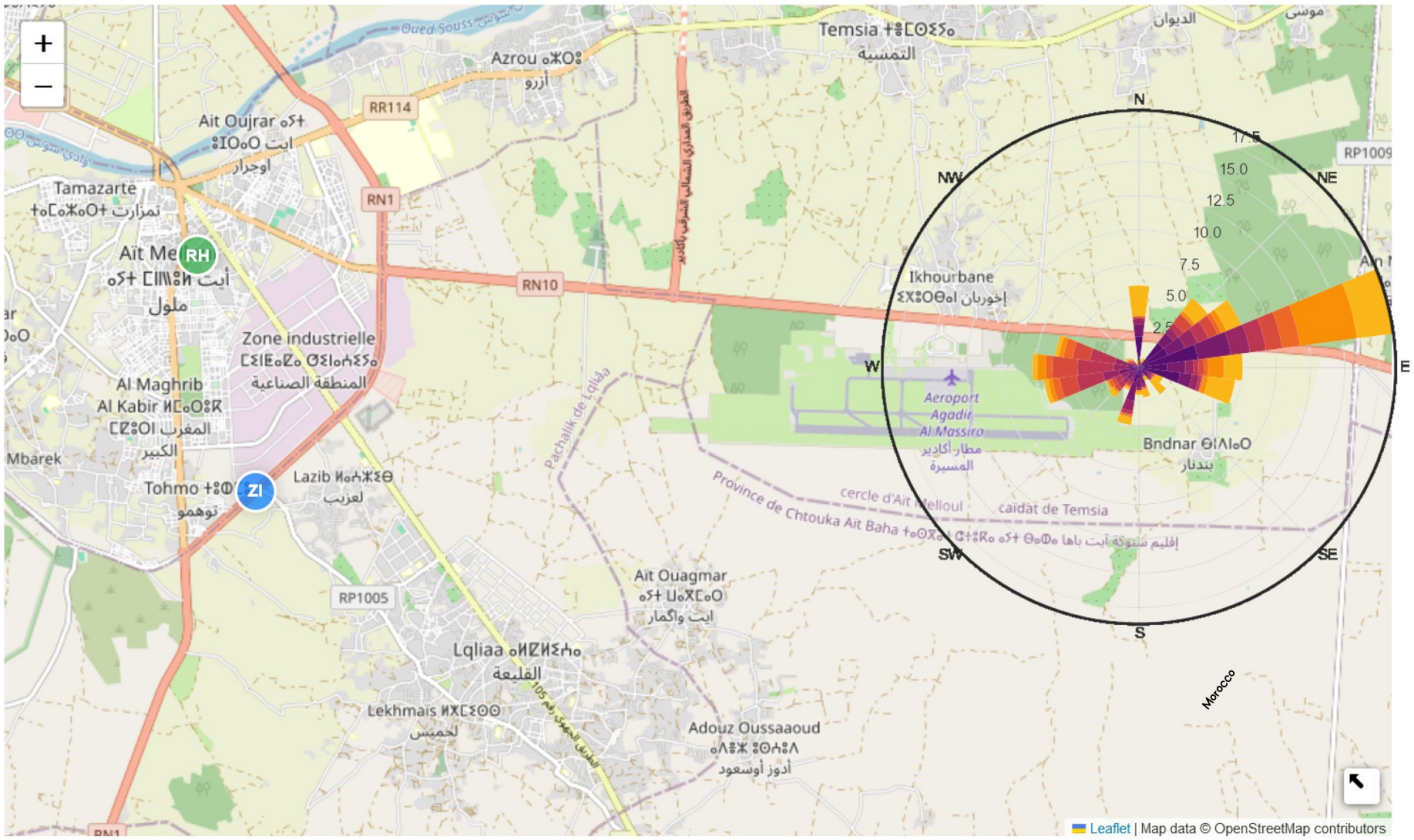
Wind rose of the wind speed and direction in Ait Melloul. Map data (C) OpenStreetMap contributors, under the Open Database License (ODbL). This map was created using the folium library in Python.

A wind rose diagram (Fig 5) provides a comprehensive representation of the predominant wind patterns observed in Ait Melloul. This diagram categorizes wind direction data into discrete sectors, crucial for determining the transport capacity and overall wind direction. According to data from the airport station, prevailing winds of moderate velocity dominate the eastern region of Ait Melloul. These winds, penetrating the industrial zone with velocities ranging from 35 to 40 km/h, likely facilitate the transportation of particles to the designated monitoring locations.

**Fig 6:**
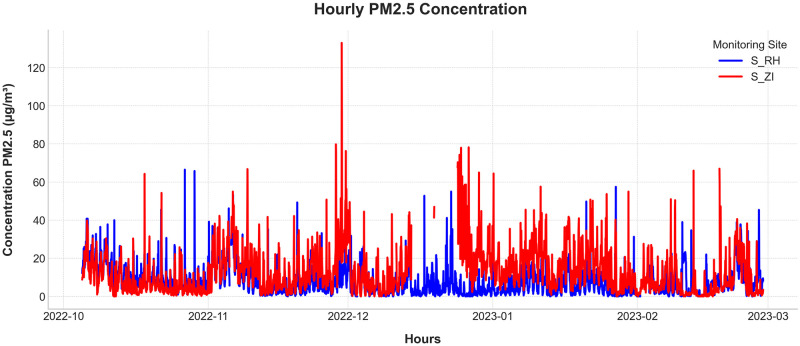
Hourly Concentration of PM_2.5_ in Ait Melloul. The figure depicts the hourly concentrations of PM_2.5_ at two monitoring sites: S_RH (blue) and S_ZI (red).

**Fig 7:**
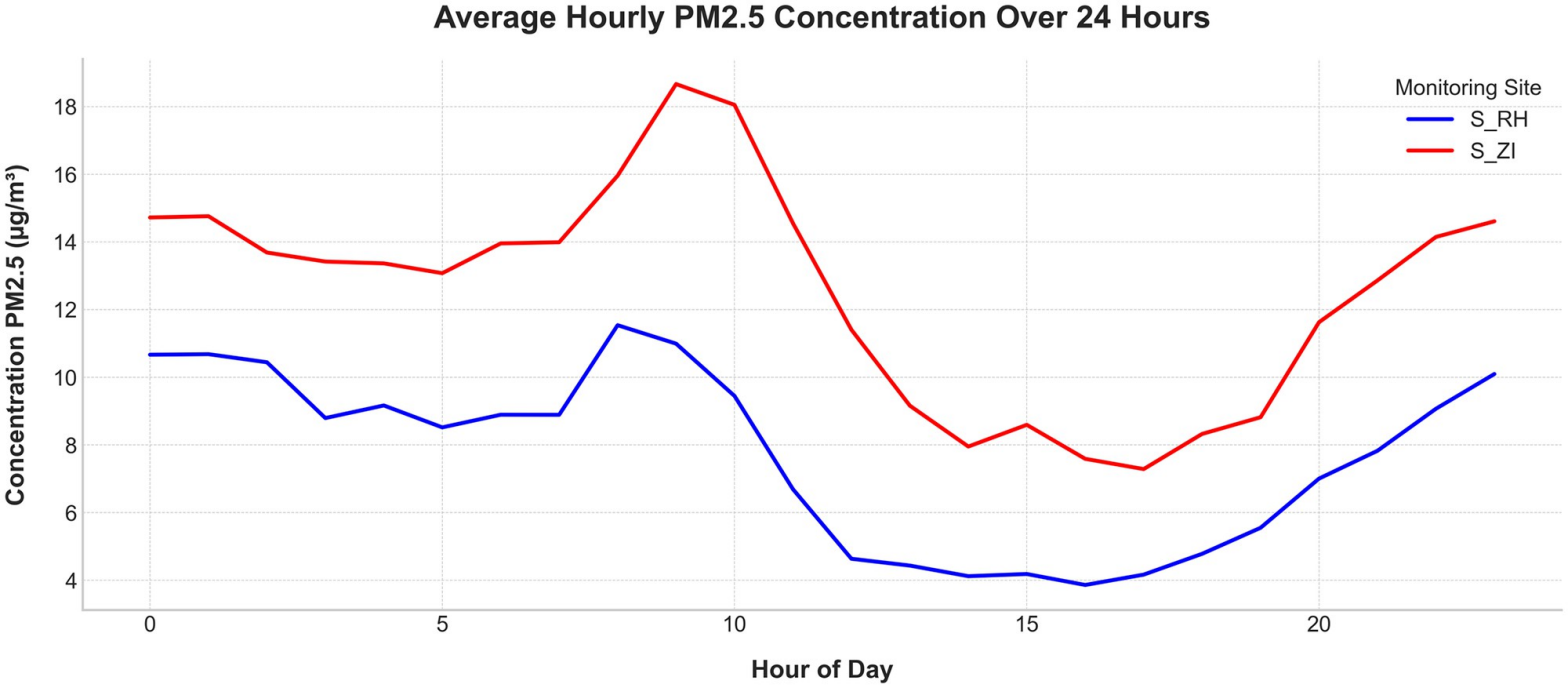
Average hourly concentration of PM_2.5_ at two monitoring stations in Ait Melloul.

Time plots are indispensable for the preliminary exploration of time-series data, facilitating the identification of trends, seasonality, anomalies, and disruptions. Such insights are crucial for selecting the most appropriate forecasting methods. Figs 6 and 7 present various graphical representations of the data utilized in this study.

Figs 6 and 7 illustrate the hourly and average hourly concentrations of PM_2.5_ recorded from October 2022 to February 2023 at two monitoring sites: S_RH (blue) and S_ZI (red). The data reveal that the distribution of PM_2.5_ does not follow a linear trend, underscoring the complexity of urban air pollution dynamics. The relatively stable variance over time suggests that the series is stationary.

Significant outliers are evident, including a pronounced peak in PM_2.5_ levels at station S_ZI on December 30th, indicative of transient pollution events. The presence of missing data, as shown in the figure, highlights the challenges associated with low-cost sensors. These gaps were addressed through interpolation during the prediction phase but are retained in the figure to emphasize potential data quality concerns.

Temporal variations in pollutant concentrations are clearly observed, with station S_ZI, located in the industrial zone, generally exhibiting higher PM_2.5_ levels compared to station S_RH, situated in a residential area. This spatial disparity underscores the influence of local emission sources, such as industrial activities and vehicular traffic, on air quality.

The average hourly PM_2.5_ concentrations exhibit discernible peaks and troughs throughout the day, with notable discrepancies between the two stations. The highest concentrations of PM_2.5_ are typically observed between 8:00 and 9:00 a.m., aligning with morning traffic and industrial activities. Station S_ZI consistently records higher PM_2.5_ levels compared to S_RH, indicating a greater influence of local emission sources. This analysis underscores the importance of understanding temporal variations in pollutant levels for effective air quality management and forecasting.

**Fig 8:**
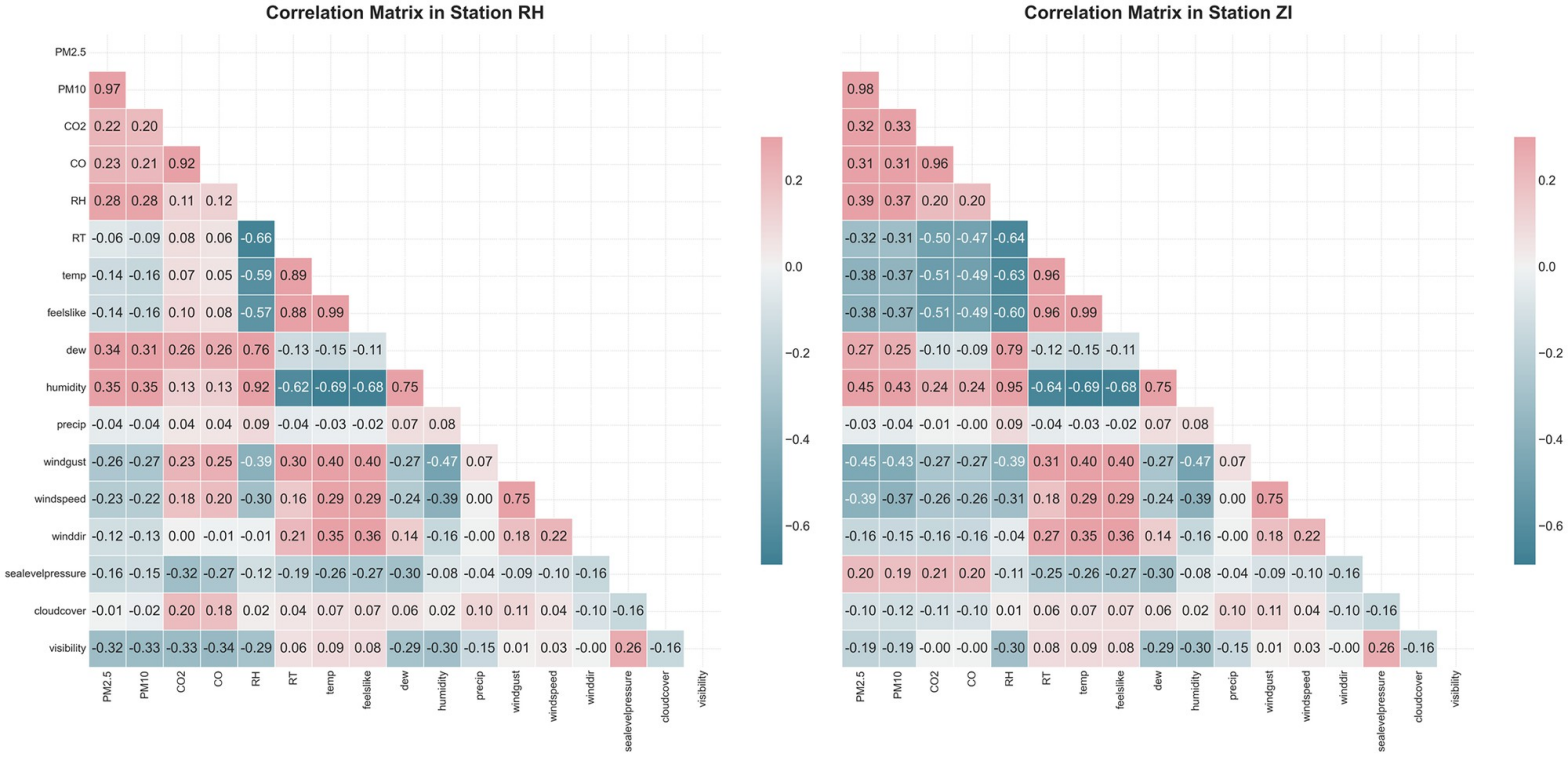
Correlation between PM_2.5_ levels and environmental parameters at two monitoring sites.

Fig 8 illustrates the correlations between PM_2.5_ levels and various environmental parameters at two distinct monitoring sites. The correlation matrices provide a comprehensive view of the relationships between PM_2.5_ and other measured variables, highlighting both positive and negative associations.

A strong positive correlation is observed between PM_2.5_ and PM_10_ levels at both sites, indicating that these two particulate matter sizes often increase and decrease together. This is due to their common sources, such as vehicular emissions, industrial activities, construction projects, and natural sources like dust and pollen. Despite their similar properties, PM_2.5_ particles are smaller and can penetrate deeper into the respiratory system, posing different health risks compared to PM_10_. To enhance the accuracy of our machine learning models, PM_10_ was excluded to avoid redundancy and improve performance.

Positive correlations are also evident between PM_2.5_ and other variables such as humidity, dew point, relative humidity (RH), carbon monoxide (CO), and carbon dioxide (CO_2_). These correlations, although weaker than that with PM_10_, suggest that increases in these parameters are associated with higher PM_2.5_ levels.

Conversely, several environmental factors exhibit negative correlations with PM_2.5_, including wind gusts, visibility, wind speed, temperature, “feels like” temperature, and sea level pressure. These negative correlations imply that increases in these factors generally correspond to decreases in PM_2.5_ concentrations. For example, higher wind speeds and gusts can disperse particulate matter, reducing its concentration in the air.

The strength of these correlations varies between the two monitoring stations. Generally, the industrial zone (S1) shows stronger correlations compared to the residential area (S2), indicating that local environmental conditions and sources of pollution significantly influence these relationships. Understanding these correlations is crucial for effective air quality management and developing predictive models for PM_2.5_ concentrations.

## 5 Predictive modeling

### 5.1 Data preprocessing

This study examines and contrasts the effectiveness and efficiency of six machine learning models for predicting PM_2.5_ concentrations. Initially, data preprocessing was undertaken, which involved the removal of outliers and the imputation of missing values. After executing an exploratory data analysis, we set up three experimental scenarios for PM_2.5_ prediction: 1) utilizing the full dataset, 2) selecting important features with the mRMR method, and 3) replicating the second scenario using LightGBM-RFE. For each scenario, we built machine learning models using the training data and assessed their predictive accuracy based on various metrics. Each experiment incorporated a range of machine learning algorithms including linear models, decision trees (DT), gradient boosting regression (GBR), support vector regression (SVR), and ensemble methods.

The detailed steps of the proposed workflow are presented in Algorithm 1.

**Algorithm 1**: Proposed Workflow for PM_2.5_ Prediction

**Input**: Meteorological Data, Historical Data from Low-cost IoT Sensors

**Output**: Predicted PM_2.5_ Concentrations

**1 begin**

  // Data Preprocessing

**2**  Perform Data Cleaning: Remove outliers and impute missing values;

**3**  Conduct Exploratory Data Analysis (EDA);

  // Feature Selection

**4**  **Scenario 1**: Use the full dataset;

**5**  **Scenario 2**: Select important features using mRMR method;

**6**  **Scenario 3**: Select important features using LightGBM-RFE method;

  // Model Building and Evaluation

**7**  Split data into Training (70%) and Testing (30%) sets;

**8**  Train models using DT, GBR, SVR, XGBoost, and LightGBM;

**9**  Optimize models using Bayesian optimization with 5-fold cross-validation;

**10**  Evaluate models using MAE, RMSE, and R^2^ on the test set;

#### 5.1.1 Data outlier

**Fig 9:**
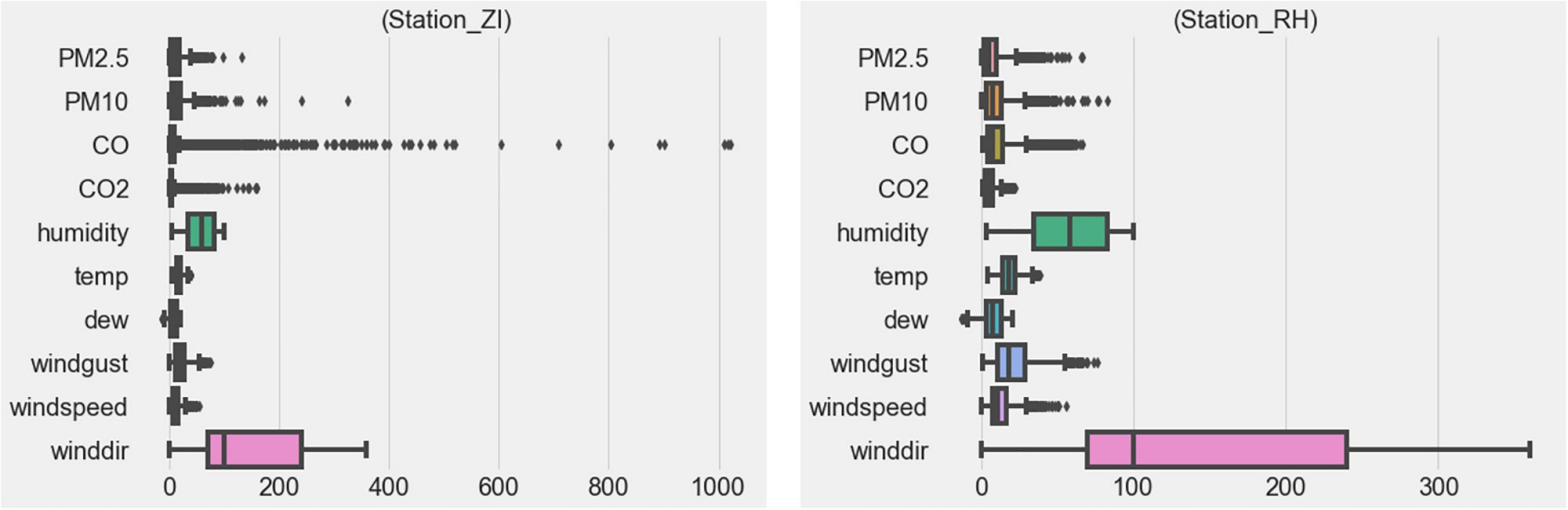
Boxplot of hourly mean air pollution levels in S1 and S2.

The dataset contains measurements with outliers, likely caused by sensor malfunctions. Cleaning the data to remove outliers and fill in missing values is crucial for improving data quality and model accuracy [52]. We used the Interquartile Range (IQR) technique to detect outliers, defined as values below *QL* − 1.5 × *IQR* or above *QU* + 1.5 × *IQR*, where *QU* and *QL* represent the upper and lower quartiles, respectively. Fig 9 shows the boxplot of hourly mean air pollution levels and meteorological factors during the study period in S1 and S2.

Outliers were treated as missing data and filled using an imputation method.

#### 5.1.2 Data imputation

Missing data is a prevalent issue in real-world datasets, including those related to air pollutant measurements. Factors such as sensor malfunctions, incorrect data recording, power outages, and data acquisition errors contribute significantly to this problem [53, 54]. These missing values can adversely affect study outcomes and the effective operation of public services related to air quality. Various methods are available to address missing values [55]. In air pollution datasets, missing values often occur in long consecutive periods due to sensor malfunctions or in short gaps resulting from routine maintenance or temporary power outages [56].

**Fig 10:**
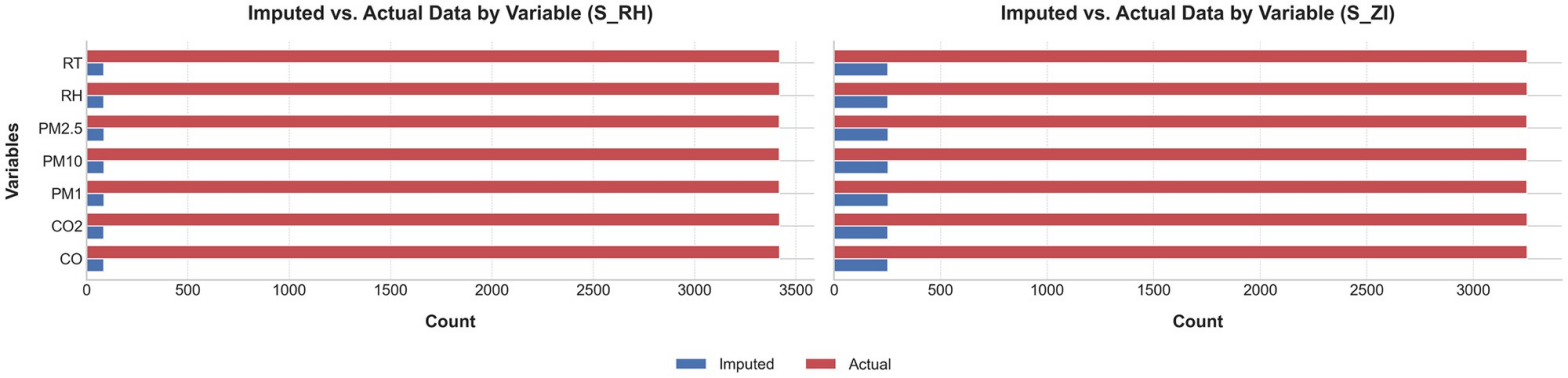
Comparison of imputed data: Actual vs. Imputed dataset in S1 and S2.

In this study, the dataset contains 1% to 5% missing values across all variables. We used the K-nearest neighbors (KNN) technique [57] to address these gaps. KNN leverages existing data to find and assign the most similar values based on the *k* nearest neighbors. Fig 10 shows the comparison between the original and imputed datasets.

#### 5.1.3 Feature selection

The dataset includes many features, some of which are irrelevant and non-essential, negatively impacting regression accuracy and increasing processing time. Thus, selecting the most relevant features is crucial.

We evaluated two methods for feature selection: LightGBM-RFE and mRMR. LightGBM-RFE excels in capturing complex, non-linear relationships through its tree-based approach, effectively handling feature interactions. mRMR focuses on selecting essential features that maximize relevance to the target variable while minimizing redundancy, using mutual information measures.

Our objective was to determine which method yields the best performance for our dataset. The following sections detail the methodologies of LightGBM-RFE and mRMR, and the rationale behind their selection for this study.

#### 5.1.4 LightGBM-RFE

LightGBM, introduced by Ke et al. in 2017 [30], is a gradient boosting framework that builds decision trees for regression and classification tasks. It is known for its efficiency in training time and memory usage while maintaining high prediction accuracy. LightGBM employs innovative techniques such as gradient one-sided sampling (GOSS) and exclusive feature bundling (EFB). Additionally, it utilizes the histogram algorithm and a leaf growth strategy with a depth limit to optimize memory consumption and prevent overfitting.

Recursive Feature Elimination (RFE) is a wrapper algorithm for feature selection, introduced by Guyon et al. [58], and it has shown significant success in various fields, including gene selection and air pollution studies. RFE iteratively reduces the feature set size by eliminating the least important features based on their importance ranking, which is recalculated at each iteration using a specific underlying algorithm.

In this study, we integrate RFE with LightGBM (LightGBM-RFE) to enhance the model’s predictive performance by selecting the most relevant features. The procedure for LightGBM-RFE is as follows:

1. **Initialize the model**: Set the desired number of features to select and specify the LightGBM model configuration. Let *n* be the total number of features, and *k* be the desired number of features to select.
2. **Train the initial model**: Train the LightGBM model using the full set of *n* features in the dataset. Let **X** = [*x*_1_, *x*_2_, …, *x*_*n*_] be the feature matrix and **y** be the target vector.
3. **Compute feature importance**: Calculate the importance of each feature based on the trained LightGBM model. The feature importance *I*(*x*_*i*_) can be defined as the total gain or split improvement brought by *x*_*i*_ across all trees in the model.
  $$I\left(x_{i}\right)=\sum_{t=1}^{T}\sum_{j\in S_{t}}\Delta I_{t}\left(j\right),$$
  where *T* is the total number of trees, $S_{t}$ is the set of splits in tree *t*, and Δ*I*_*t*_(*j*) is the improvement in the loss function due to the split on feature *x*_*j*_.
4. **Eliminate least important features**: Identify and eliminate the least important features based on their importance ranking. Let F be the set of remaining features. Remove the feature *x*_*min*_ with the lowest importance: $F\leftarrow F\backslash \{x_{min}\}$.
5. **Retrain the model**: Retrain the LightGBM model using the reduced set of features F.
6. **Repeat the process**: Repeat the process of feature elimination and model retraining until the desired number of features *k* is selected.
7. **Train the final model**: Train the final LightGBM model using the selected subset of *k* features.

The integration of RFE with LightGBM provides a powerful method for feature selection, leveraging the efficiency and accuracy of LightGBM to identify the most relevant features while iteratively reducing the feature set to enhance model performance and reduce overfitting.

#### 5.1.5 mRMR (Minimum Redundancy Maximum Relevance)

Minimum Redundancy Maximum Relevance (mRMR) [59] is a feature selection method designed to select a subset of features that are both highly relevant to the target variable and minimally redundant among themselves. Relevance is defined as the degree to which a feature is related to the target variable, while redundancy is the degree to which features are correlated with each other.

The mRMR algorithm operates in two main stages: calculating relevance and redundancy. Relevance is measured using mutual information, which quantifies the statistical dependence between two variables. Redundancy is measured using the Pearson correlation coefficient between pairs of features. The goal of mRMR is to maximize relevance and minimize redundancy, ensuring the selected features are the most representative of the target variable without being overly correlated with each other.

The steps of the mRMR algorithm are as follows:

1. **Initialization**: Begin with an empty set of selected features S. Calculate the relevance score for each feature *x*_*i*_ with respect to the target variable *y* using mutual information *I*(*x*_*i*_; *y*):
  $$I\left(x_{i};y\right)=\int_{x_{i}}\int_{y}p\left(x_{i},y\right)\text{log}(\frac{p(x_{i},y)}{p\left(x_{i}\right)p\left(y\right)})dx_{i}dy,$$
  where *p*(*x*_*i*_, *y*) is the joint probability distribution of *x*_*i*_ and *y*, and *p*(*x*_*i*_) and *p*(*y*) are the marginal probability distributions.
2. **Maximum Relevance**: Select the feature *x*_*i*_ with the highest relevance score and add it to the set S:
  $$S\leftarrow S\cup \left\{x_{i}\right\},\;\text{where}\;x_{i}=\text{arg}\underset{x_{j}\in F\backslash S}{\text{max}}\;I\left(x_{j};y\right).$$
3. **Minimum Redundancy**: Calculate the redundancy between each remaining feature *x*_*j*_ and the features already in S using average mutual information:
  $$R\left(x_{j},S\right)=\frac{1}{\left|S\right|}\sum_{x_{i}\in S}I\left(x_{j};x_{i}\right).$$
  Select the feature *x*_*j*_ that maximizes the difference between relevance and redundancy:
  $$S\leftarrow S\cup \left\{x_{j}\right\},\;\text{where}\;x_{j}=\text{arg}\underset{x_{k}\in F\backslash S}{\text{max}}\;(I\left(x_{k};y\right)-R\left(x_{k},S\right)).$$
4. **Iteration**: Repeat steps 2 and 3 until the desired number of features is selected or another stopping criterion is met.
5. **Final Subset**: The final subset S consists of the features selected during the iterative process.

By balancing relevance and redundancy, the mRMR algorithm ensures that the selected features provide maximum information about the target variable while minimizing overlap, leading to a more efficient and interpretable feature set.

### 5.2 Pipeline and performance criteria

Time series prediction is crucial for estimating future information using past and present data. This study employs a “next-hour prediction” methodology based on supervised learning principles. The initial dataset is divided into historical datasets (*t* = −24, *t* = −23, …, *t* = 0) and a dataset for the following hour (*t* = 1). These datasets are then combined to create a comprehensive time-series dataset for forecasting future values (*t* = 1, *t* = 2, …, *t* = *n*).

In constructing the machine learning models, the dataset was first organized and then split into two subsets: 70% for training and 30% for evaluation. For LightGBM, data normalization was unnecessary, but preprocessing, including normalization, was essential for other machine learning approaches like Support Vector Regression. The normalization formula used is as follows:

$$\begin{array}{c} X_{norm}=\frac{X_{i}-X_{i,min}}{X_{i,max}-X_{i,min}} \end{array}$$ (1)

Where *X*_*norm*_, *X*_*i*_, *X*_*i*,*min*_, and *X*_*i*,*max*_ represent the normalized value, the actual value, the minimum value, and the maximum value, respectively.

The optimal hyperparameters for the SVR, XGBoost, and LightGBM models were determined using Bayesian Optimization. After running the algorithm for 100 iterations to obtain the hyperparameter values, a final model was trained and tested.

#### 5.2.1 Evaluation metrics

The performance of the proposed model is evaluated using several metrics to provide a comprehensive assessment. The primary metrics used are RMSE (Root Mean Square Error), MAE (Mean Absolute Error), and *R*^2^ (coefficient of determination).

*5.2.1.1 Root Mean Square Error (RMSE)*. RMSE measures the square root of the average squared differences between observed and predicted values, making it sensitive to outliers.

$$\text{RMSE}=\sqrt{\frac{1}{n}\sum_{i=1}^{n}{\left(y_{i}-{\hat{y}}_{i}\right)}^{2}}$$

*5.2.1.2 Mean Absolute Error (MAE)*. MAE measures the average absolute differences between observed and predicted values.

$$\text{MAE}=\frac{1}{n}\sum_{i=1}^{n}\left|y_{i}-{\hat{y}}_{i}\right|$$

*5.2.1.3 Coefficient of Determination* (*R*^2^). *R*^2^ indicates the proportion of variance in the dependent variable predictable from the independent variables.

$$R^{2}=1-\frac{\sum_{i=1}^{n}{\left(y_{i}-{\hat{y}}_{i}\right)}^{2}}{\sum_{i=1}^{n}{\left(y_{i}-\bar{y}\right)}^{2}}$$

*5.2.1.4 Additional metrics*. To ensure robust evaluation, additional metrics are considered:

- **Mean Squared Error (MSE)**: Measures the average of the squared differences.
  $$\text{MSE}=\frac{1}{n}\sum_{i=1}^{n}{\left(y_{i}-{\hat{y}}_{i}\right)}^{2}$$
- **Explained Variance Score**: Measures the proportion of variance explained by the model.
  $$\text{Explained}\;\text{Variance}=1-\frac{\text{Var}(y-\hat{y})}{\text{Var}(y)}$$
- **Median Absolute Error (MedAE)**: Measures the median of the absolute errors.
  $$\text{MedAE}=\text{median}(|y_{1}-{\hat{y}}_{1}|,|y_{2}-{\hat{y}}_{2}|,\ldots ,|y_{n}-{\hat{y}}_{n}|)$$

Using multiple evaluation metrics allows for a comprehensive assessment of model performance.

## 6 Results and discussion

The effectiveness of a machine learning model heavily depends on the selection of hyperparameters. In this study, Bayesian optimization combined with k-fold cross-validation was employed to optimize these hyperparameters. Specifically, the dataset was partitioned into *k* = 5 subsets for cross-validation, enhancing the model’s ability to generalize by training on different subsets and testing on the remaining ones.

Our experiment aimed to assess the impact of historical PM_2.5_ data on forecasting future concentrations. Three scenarios were evaluated: (1) forecasting without feature selection, (2) with feature selection using the mRMR algorithm, and (3) with feature selection using the LightGBM-RFE algorithm. Various regression models, including Linear Regression, Random Forest, XGBoost, Gradient Boosting, and SVR, were examined. Performance metrics such as *R*^2^, RMSE, MAE, MSE, MedAE, and Explained Variance Score were used for evaluation.

To illustrate the different sets of features used for PM_2.5_ prediction in the ZI dataset, we have summarized the subsets selected by the All, mRMR, and LightGBM-RFE feature selection methods in Table 2.

**Table 2:** Feature sets from ZI dataset.

Feature Selection Method	Features
All	PM2.5, CO2, CO, RH, RT, temp, feelslike, dew, humidity, precip, windgust, windspeed, winddir, sealevelpressure, cloudcover, visibility
mRMR	PM2.5, CO2, windgust, temp, feelslike, sealevelpressure, humidity
LightGBM-RFE	PM2.5, CO2, RH, RT, windgust, winddir, humidity

This table presents the different sets of features used for PM_2.5_ prediction in the ZI dataset, showcasing the subsets selected by the All, mRMR, and LightGBM-RFE feature selection methods.

### 6.1 Primary metrics analysis

Table 3 shows the performance of the models using primary metrics (*R*^2^, RMSE, and MAE) across three forecasting horizons (1H, 2H, and 3H) and under different feature selection methods (ALL, mRMR, LightGBM-RFE).

**Table 3:** Performance of machine learning models on IZ dataset (Primary metrics).

Model	ALL			mRMR			LightGBM-RFE		
	R2	RMSE	MAE	R2	RMSE	MAE	R2	RMSE	MAE
**1 Hour Forecast**									
Linear Regression	0.590	5.859	4.276	0.697	5.063	3.547	0.708	5.033	3.501
Random Forest	0.718	4.857	3.382	0.712	4.925	3.425	0.724	4.831	3.292
XGBoost	0.714	4.986	3.413	0.725	4.859	3.426	0.732	4.782	3.326
Gradient Boosting	0.634	5.581	4.175	0.633	5.480	4.192	0.653	5.389	4.115
SVR	0.685	5.156	3.701	0.693	5.101	3.483	0.704	4.988	3.462
LightGBM	0.723	4.850	3.448	0.726	4.829	3.406	0.747	4.559	3.236
**2 Hour Forecast**									
Linear Regression	0.532	6.295	4.632	0.647	5.505	3.892	0.643	5.516	3.916
Random Forest	0.652	5.382	3.919	0.657	5.416	3.918	0.660	5.316	3.799
XGBoost	0.661	5.538	3.963	0.666	5.308	3.803	0.662	5.271	3.876
Gradient Boosting	0.532	6.276	4.744	0.510	6.426	4.889	0.482	6.617	5.070
SVR	0.648	5.539	3.980	0.645	5.480	3.791	0.650	5.449	3.891
LightGBM	0.679	5.238	3.830	0.678	5.311	3.789	0.676	5.237	3.780
**3 Hour Forecast**									
Linear Regression	0.485	6.608	3.599	0.606	5.800	4.162	0.592	5.833	4.221
Random Forest	0.600	5.816	4.330	0.594	5.856	4.330	0.611	5.743	4.211
XGBoost	0.610	5.733	4.228	0.624	6.653	4.090	0.633	5.592	4.098
Gradient Boosting	0.502	6.463	4.914	0.488	5.605	5.049	0.441	6.885	5.250
SVR	0.602	5.789	4.182	0.602	5.777	4.064	0.603	5.763	4.233
LightGBM	0.627	5.601	4.111	0.624	5.642	4.041	0.644	5.522	3.995

This table summarizes the performance of various machine learning models across three forecasting horizons (1H, 2H, 3H) and under different feature selection methods (ALL, mRMR, LightGBM-RFE). The metrics used for evaluation include *R*^2^, RMSE, and MAE.

For the 1-hour forecast, the LightGBM model exhibited superior performance with an *R*^2^ of 0.747, RMSE of 4.559, and MAE of 3.236 when using the LightGBM-RFE method. This strong alignment with observed data underscores its reliability for short-term predictions. The Random Forest and XGBoost models also performed well, particularly with the LightGBM-RFE method, achieving *R*^2^ values above 0.72.

As the forecasting horizon extended to 2 hours, a slight decline in performance was observed, which is expected due to the increased complexity of predictions. Nevertheless, the LightGBM model remained the top performer with an *R*^2^ of 0.678, RMSE of 5.237, and MAE of 3.780. Random Forest and XGBoost maintained competitive performance levels, reflecting their robustness.

For the 3-hour forecast, the LightGBM model continued to lead with an *R*^2^ of 0.644, RMSE of 5.522, and MAE of 3.995. Although accuracy decreased with the longer forecast horizon, LightGBM’s predictive power remained evident compared to other models.

Linear Regression consistently showed the lowest performance across all horizons and feature selection methods, with *R*^2^ values below 0.61 for the 3-hour forecast, indicating its limitations for complex air quality prediction tasks.

These results highlight the significant impact of feature selection on model performance, with the LightGBM-RFE method consistently enhancing predictive accuracy across all models and forecasting horizons.

### 6.2 Additional metrics analysis

Table 4 provides additional performance metrics (MSE, MedAE, and Explained Variance) for the same models and scenarios. These metrics offer deeper insights into error distribution and variance explanation.

**Table 4:** Performance of machine learning models on IZ dataset (Additional metrics).

Model	ALL			mRMR			LightGBM-RFE		
	MSE	MedAE	Exp. Var.	MSE	MedAE	Exp. Var.	MSE	MedAE	Exp. Var.
**1 Hour Forecast**									
Linear Regression	34.05	3.20	0.60	25.63	2.48	0.69	25.34	2.35	0.70
Random Forest	23.74	2.40	0.73	23.95	2.40	0.72	23.28	2.15	0.73
XGBoost	23.80	2.43	0.72	23.50	2.48	0.72	22.83	2.30	0.73
Gradient Boosting	30.95	3.83	0.68	31.30	3.34	0.65	29.04	3.34	0.68
SVR	26.68	2.63	0.68	26.02	2.31	0.70	24.76	2.30	0.71
LightGBM	24.48	2.73	0.72	23.20	2.43	0.73	22.19	2.29	0.74
**2 Hour Forecast**									
Linear Regression	39.59	3.49	0.54	30.27	2.72	0.64	30.32	2.72	0.65
Random Forest	28.91	2.96	0.67	29.52	2.96	0.66	28.10	2.69	0.67
XGBoost	27.80	2.86	0.68	28.11	2.84	0.67	26.74	2.67	0.69
Gradient Boosting	35.98	4.23	0.62	35.87	3.76	0.60	34.37	3.66	0.62
SVR	30.56	2.80	0.64	30.00	2.52	0.65	29.26	2.83	0.66
LightGBM	28.82	3.07	0.67	28.07	2.78	0.67	27.35	2.83	0.68
**3 Hour Forecast**									
Linear Regression	43.57	3.73	0.49	33.60	3.01	0.60	33.98	3.03	0.61
Random Forest	33.74	3.44	0.61	34.00	3.42	0.61	32.96	3.23	0.62
XGBoost	31.71	3.18	0.63	31.87	3.08	0.62	30.51	2.96	0.64
Gradient Boosting	40.92	4.53	0.56	41.07	4.16	0.55	39.41	4.01	0.57
SVR	33.53	3.00	0.60	33.30	2.77	0.61	33.17	3.23	0.62
LightGBM	32.50	3.30	0.62	31.98	3.05	0.62	31.28	3.16	0.63

This table summarizes the performance of various machine learning models across three forecasting horizons (1H, 2H, 3H) and under different feature selection methods (ALL, mRMR, LightGBM-RFE). The metrics used for evaluation include MSE, MedAE, and Exp. Var.

For the 1-hour forecast, the LightGBM model with LightGBM-RFE achieved the lowest MSE (22.19), MedAE (2.29), and the highest Explained Variance (0.74), reaffirming its strong performance.

In the 2-hour forecast, LightGBM maintained its leading position with an MSE of 27.35, MedAE of 2.83, and Explained Variance of 0.68, demonstrating robustness as the prediction horizon extends.

For the 3-hour forecast, LightGBM continued to perform well with an MSE of 31.28, MedAE of 3.16, and Explained Variance of 0.63, indicating its consistent ability to handle longer-term forecasts.

The additional metrics support the primary metrics findings, showing that LightGBM, particularly with the LightGBM-RFE feature selection method, consistently delivers high accuracy and reliability in PM_2.5_ forecasting.

**Fig 11:**
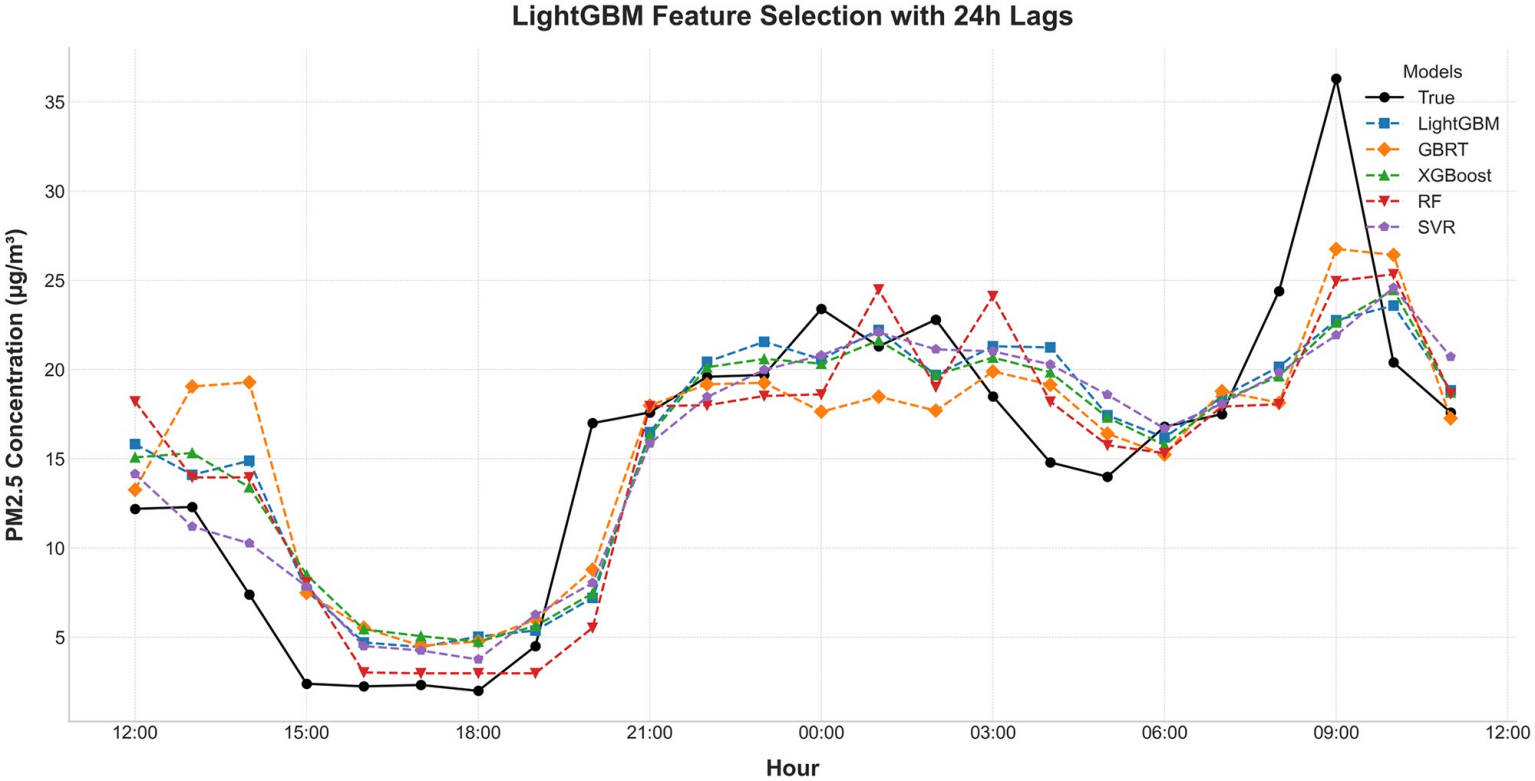
Observed and predicted PM_2.5_ concentrations.

**Fig 12:**
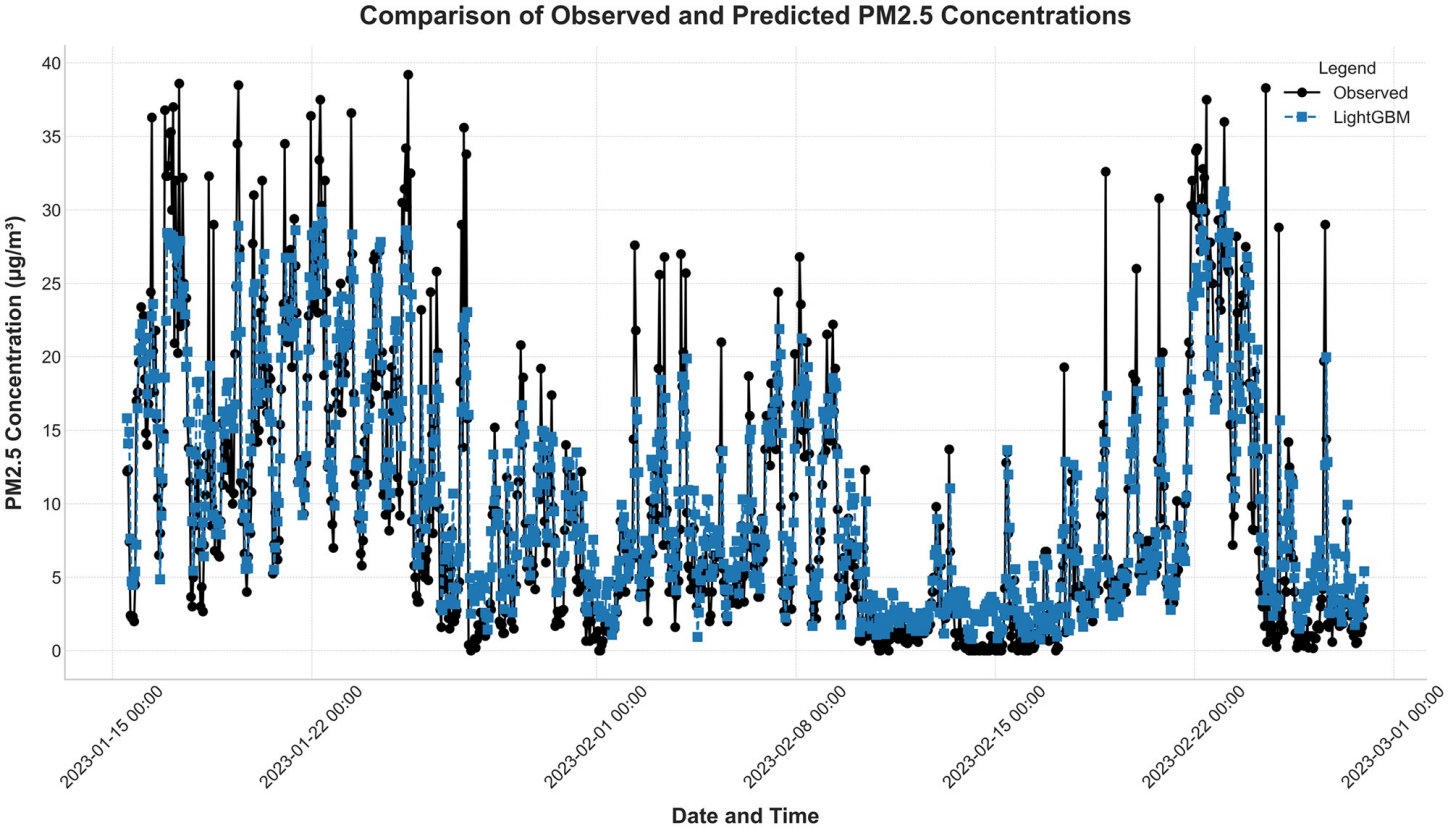
Fit curve of PM_2.5_ real value and predicted value using the LightGBM model.

Figs 11 and 12 further illustrate the effectiveness of the LightGBM model. The first figure shows the observed and predicted PM_2.5_ concentrations, demonstrating the model’s capability to closely follow actual trends. The second figure presents the fit curve of the PM_2.5_ real values and the predicted values using the LightGBM model, indicating a good degree of alignment and minimal deviations.

### 6.3 Explainable AI with SHAP

The SHAP (SHapley Additive exPlanations) method was employed to convert the typically opaque machine learning model into an interpretable model that illustrates the influence of each feature on the prediction of PM_2.5_ levels. This innovative approach, based on the Shapley values concept from cooperative game theory as proposed by Lloyd Shapley [60], has been adapted to machine learning by Lundberg and Lee [61] to offer a unified framework for model interpretation. The scores are calculated based on the contributions of individual features, offering insights into how each feature affects the model’s output. The utilization of SHAP brings several notable benefits, including enhanced transparency, deeper insights into the model’s decision-making process, and more informed and accurate interpretations of predictive outcomes.

The key benefits of employing the SHAP method in our study are:

1. Investigating the relationship between particular characteristics and forecasting, enhancing our understanding of how environmental and anthropogenic factors influence PM_2.5_ levels.
2. Analyzing factors influencing predictions to obtain more comprehensive and nuanced understandings, facilitating the identification of significant predictors and their interactions within the model.
3. Unraveling the intricacies of the machine learning opaque system, making the decision-making process of complex models transparent and comprehensible for both researchers and practitioners.

**Fig 13:**
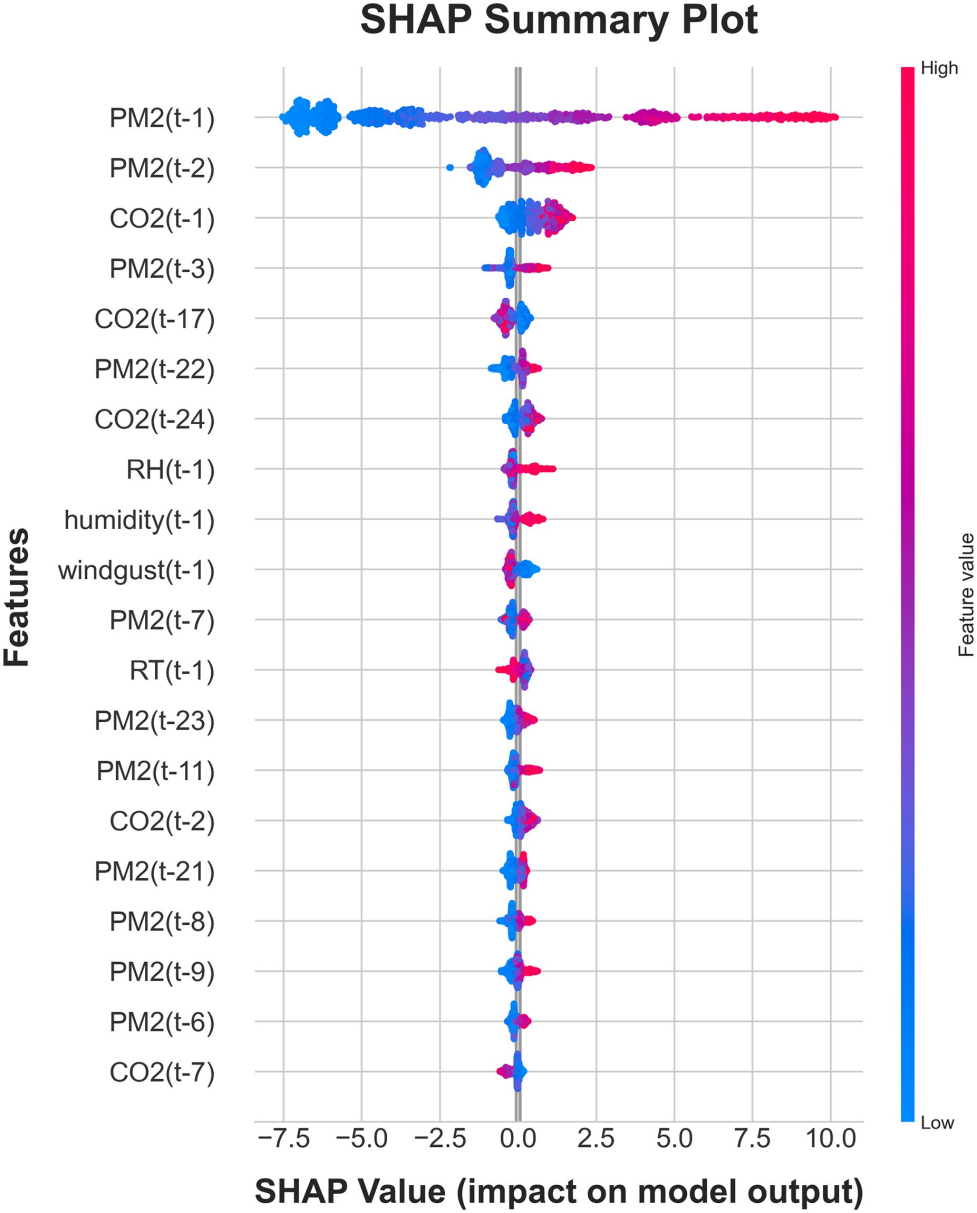
Feature ranking using the SHAP values for the LightGBM model.

The proposed approach involved the integration of LightGBM with SHAP. As depicted in Fig 13, the color red represents the maximum value for each selected feature on its unit scale, while the color blue indicates the minimum value.

Upon analysis of Fig 13, it is observed that the curves representing the genuine values and the predictions produced by the LightGBM model demonstrate a comparable pattern and display a good degree of alignment. This finding suggests that the model put forward in this study effectively encompasses the temporal and spatial fluctuations of PM_2.5_, facilitating reasonably precise forecasts of PM_2.5_ concentrations.

**Fig 14:**
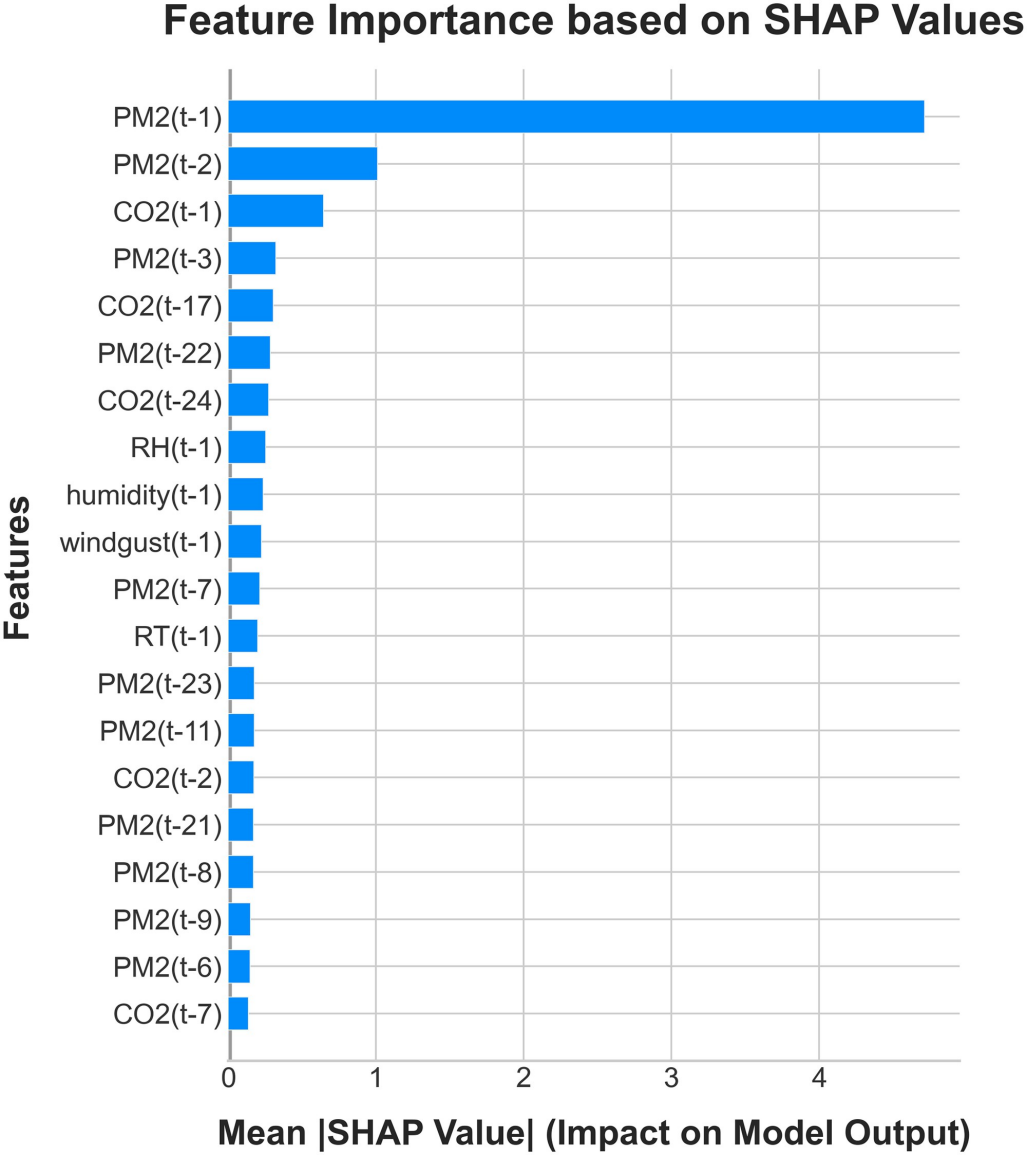
Feature importance plot derived from the LightGBM model.

Feature significance plots were constructed for the LightGBM model using the SHAP values. The most significant features, arranged in descending order based on their respective impacts, are presented in Fig 14. The five features that exhibited the greatest influence were PM_2.5_ at time *t* − 1, PM_2.5_ at time *t* − 2, CO_2_ at time *t* − 1, and PM_2.5_ at time *t* − 3.

To enhance the understanding of the effects of feature importance on the model’s output, we have included a SHAP summary plot in Fig 13. The plot demonstrates that features with higher SHAP values are more likely to have a significant impact on the predictions generated by the LightGBM model. The SHAP values, denoted by red dots, signify an augmentation in prediction, whereas the blue dots imply a reduction in prediction.

The variable PM_2.5_(*t* − 1) denotes the level of PM_2.5_ concentration observed one hour prior to the forecasted date. It is evident that a majority of the blue sample points are located within the left half of the territory, while a majority of the purple sample points are situated within the right half of the region. This implies that the feature results in a reduction of the predicted concentration when the PM_2.5_ concentration one hour prior is lower, and an increase in the predicted concentration when the PM_2.5_ concentration one hour prior is higher. The scenario involving feature PM_2.5_(*t* − 2) bears resemblance to that of feature PM_2.5_(*t* − 1), with a notable distinction in the magnitude of importance, where the peak of significance for feature PM_2.5_(*t* − 1) is higher.

The distinction between the feature wind gust and the features PM_2.5_(*t* − 1) and PM_2.5_(*t* − 2) is evident. The observation reveals that a majority of the blue sample points inside this feature row are situated in the right zone, whilst the purple sample points are predominantly concentrated in the left part of the zone. This implies that when making predictions about the concentration of PM_2.5_, the inclusion of the feature results in an increase in the projected concentration of PM_2.5_, whereas the exclusion of the feature leads to a drop in the predicted concentration of PM_2.5_.

### 6.4 Comparison, analysis, and implications of results

The Light Gradient Boosting Regressor consistently exhibits superior performance compared to other models across all time forecasting scenarios. It attains top ratings in *R*^2^, RMSE, MAE, MSE, MedAE, and Explained Variance Score. For instance, the 1-hour forecast showed a commendable *R*^2^ of 0.747, signifying strong alignment with the provided data and minimal variance from actual values. The RMSE test result of 4.559 and MAE test value of 3.236 indicate precise forecasts with minimum variance from the actual values.

The Gradient Boosting Regressor and Support Vector Regression (SVR) models also demonstrated robust performance across all measures. These models consistently demonstrated reliable performance across various time intervals, indicating their potential for accurate air quality predictions. In contrast, Linear Regression regularly demonstrated inferior performance compared to the other models, with lower scores across all evaluation metrics, indicating a comparatively poorer alignment and increased prediction errors.

The selection of the optimal model ultimately depends on the specific demands and priorities of the analysis. Nevertheless, the results clearly indicate that the Light Gradient Boosting Regressor is a highly favorable option due to its consistently robust performance across all time intervals. This underscores the importance of model choice and feature selection in enhancing predictive accuracy and reliability.

The implications of these results highlight the importance of advanced machine learning techniques, particularly the LightGBM model with the LightGBM-RFE feature selection method, in accurately predicting PM_2.5_ concentrations. This capability is crucial for developing effective air quality management strategies in urban environments, enabling authorities to make data-driven decisions to mitigate pollution and protect public health.

## 7 Conclusion

This study introduces an innovative architectural framework for smart city applications, centered on the integration of Artificial Intelligence of Things (AIoT) to enhance sustainability and improve quality of life. The proposed architecture consists of three key levels:

1. **Data Collection**: Sensors collect environmental data, which is transmitted to a Cloud Server (CS) via a wireless network and the MQTT (Message Queuing Telemetry Transport) protocol.
2. **Data Processing**: Data is processed using a Node-Red infrastructure and stored in InfluxDB.
3. **Data Analytics**: A centralized Data Analytics server employs machine learning algorithms for control and prediction.

We propose the use of a LightGBM model to predict PM_2.5_ levels, with data preprocessing steps including the elimination of redundant attributes, removal of outliers, and imputation of missing values. Feature selection techniques such as minimum Redundancy Maximum Relevance (mRMR) and LightGBM Recursive Feature Elimination (LightGBM-RFE) were employed to identify the most significant features, streamlining the model for optimal performance. Bayesian Optimization and 5-fold cross-validation were used to enhance the efficiency of various machine learning models, including Random Forest (RF), Gradient Boosting Regression (GBR), XGBoost, Support Vector Regression (SVR), and LightGBM, resulting in high precision.

To provide insights into the model’s predictions, we employed the SHAP (Shapley Additive Explanations) method, which helps interpret the influence of individual features on PM_2.5_ predictions. Evaluation metrics such as *R*^2^, RMSE, and MAE were used to comprehensively assess the predictive performance of the models.

The novelty of this work lies in the integration of low-cost IoT sensors with advanced machine learning techniques to create a scalable and cost-effective air quality monitoring and prediction system. By combining feature selection methods like mRMR and LightGBM-RFE with Bayesian optimization, our approach achieves high accuracy in PM_2.5_ predictions. This study is among the first to implement such a comprehensive system in a resource-limited setting like Morocco, offering a valuable framework for other developing regions facing similar challenges.

Future research will focus on integrating deep learning methodologies with machine learning algorithms to analyze a larger, more diverse air quality dataset. This expanded dataset will incorporate innovative features, further enhancing our understanding and predictive capabilities for air quality management.
